# Bone turnover after bariatric surgery

**DOI:** 10.1590/2359-3997000000279

**Published:** 2017-06-23

**Authors:** Thalita Lima Melo, Leila Froeder, Leandro da Cunha Baia, Ita Pfeferman Heilberg

**Affiliations:** 1 Universidade Federal de São Paulo São Paulo SP Brasil Disciplina de Nefrologia, Universidade Federal de São Paulo (Unifesp), São Paulo, SP, Brasil

**Keywords:** Gastric bypass, bone turnover, obesity, bone metabolism

## Abstract

**Objective:**

The aim of the present study was to evaluate parameters of bone and mineral metabolism after bariatric surgery.

**Subjects and methods:**

This sectional study included data from medical records from 61 bariatric surgery (BS) patients (minimum period of 6 months after the procedure) and from 30 class II and III obese patients as a control group (Cont), consisting of daily dietary intake of macronutrients, calcium and sodium, serum 25(OH)D and parathyroid hormone (PTH) and other biochemical serum and urinary parameters. Bone alkaline phosphatase (BAP), leptin, fibroblast growth factor-23 (FGF-23) and deoxypyridinoline (DPYD) were determined from available banked serum and urinary samples.

**Results:**

Mean body mass index (BMI), median energy, carbohydrate, protein and sodium chloride consumption were significantly lower in the BS versus Cont, but calcium and lipids were not. No significant differences were found in ionized calcium, 25(OH)D, PTH and fibroblast growth factor 23 (FGF-23) between groups. Mean serum BAP was significantly higher for BS versus Cont and had a positive correlation with time after the surgical procedure. Mean serum leptin was significantly lower and median urinary DPYD higher in BS versus Cont.

**Conclusion:**

The present study showed an increase in bone markers of both bone formation and resorption among bariatric patients up to more than 7 years after the surgical procedure, suggesting that an increased bone turnover persists even at a very long-term follow-up in such patients.

## INTRODUCTION

The prevalence of obesity has increased during the past decades representing a major public health problem. A high body mass index (BMI) has been directly associated with higher bone mineral density (BMD) given that mechanical loading under physiological conditions possesses a central role on bone mass maintenance. Therefore, it has been well accepted until recently, that obesity was associated with a reduced risk of osteoporosis and that fat mass had a similar beneficial effect on increasing bone mass (1). However, contrasting studies now show that excessive fat mass may not protect against osteoporosis (2) and that lean mass may be a more important determinant of BMD (3). Moreover, adipose tissue expresses and secretes several biologically active hormones and cytokines such as estrogen, leptin, among others, which may affect energy homeostasis and bone metabolism (4).

Bariatric surgery (BS) is an effective treatment for clinically severe obesity and Roux-en-Y gastric bypass, the most common technique, induces an important and sustained weight loss improving comorbidities. However, the procedure entails long-term complications such as nutritional deficiencies, nephrolithiasis (5), as well as other disturbances in bone and mineral metabolism, including low bone mass and increased risk of fractures after a substantial weight loss (6-9). Bone loss may be attributed to a reduction of lean body mass during the weight reduction process, previous obesity-induced hypovitaminosis D, which persists even after surgery, and malabsorption of calcium and vitamin D following the procedure (10,11). De Prisco and Levine (6) reported osteomalacia and osteoporosis among bariatric patients up to 12 years after procedure, suggesting that this population is at risk for metabolic bone disease at a long-term basis.

Previous studies that evaluated skeletal changes after bariatric surgery through dual-energy x-ray absorptiometry (DXA), quantitative computed tomography (QCT) and high-resolution peripheral computed tomography (HR-pQCT) demonstrated a decreased BMD and also increased bone turnover markers (7,9,12-15).

Therefore, bone loss is a multifactorial complication in bariatric patients although the underlying mechanisms are not fully elucidated. The aim of the present study was to evaluate parameters of bone and mineral metabolism after bariatric surgery.

## SUBJECTS AND METHODS

A sectional study was carried out by the review of 80 medical records from class II and III obese patients who had undergone bariatric surgery (BS) through Roux-en-Y (RYGB) or biliopancreatic diversion with duodenal switch (BD-DS) between 2007 and 2010 at the Universidade Federal de São Paulo. Eligible patients for inclusion were those with a minimum period of 6 months post-surgery, who had dietary information obtained through 72 hour food recalls, as well as results from serum levels of 25(OH)D and parathyroid hormone (PTH), and other biochemical serum and urinary (retrieved from 24 hour urine specimens) parameters under conditions of discontinuation of multi-vitamins and/or calcium supplementation 72 hours before collection. Serum biochemistry comprised values of creatinine, albumin and ionized calcium and urinary parameters included calcium, sodium and creatinine. This retrospective analysis was followed by a further selection of patients whose banked serum and urine samples were available for the determination of additional mineral metabolism parameters, such as fibroblast grow factor-23 (FGF-23), bone alkaline phosphatase (BAP), deoxypyridinoline (DPYD) as well as leptin. Exclusion criteria were age < 18 years old, abnormal renal function (estimated glomerular filtration rate (eGFR) by CKD-EPI creatinine equation < 60 mL/min/1.73 m^2^) and history of medical disorders known to affect bone metabolism (*e.g*., hyperthyroidism, hyperparathyroidism, renal disease, Paget’s disease, etc.).

The study was approved by the local Ethics Committee of the Institution and a written consent was obtained from all participants. The control group (Cont) consisted of data from age-matched obese patients with a body mass index [BMI] 35.0–39.9 (obesity class II) and ≥ 40 kg/m^2^ (obesity class III) waiting for bariatric surgery, whose similar parameters could be used for comparisons.

Serum BAP (Quidel, San Diego, CA), FGF-23 (Kainos Laboratories, Tokyo, Japan), fasting serum leptin (Merck Millipore, Darmstadt, Alemanha) and urinary DPYD (MicroVue DPD EIA, Quidel Corporation, San Diego, USA) were measured by ELISA.

### Statistical analyses

It was estimated that this sample size would provide 95% of power, at a significance level of 0.01, to detect changes in primary endpoints by including at least 15 patients in each group. The software G. Power 3.1.2 was used for the sample calculation (Franz Faul, University of Kiel, Germany).

Normally distributed variables are presented as mean ± standard deviation and skewed variables as median [interquartile interval]. Differences between groups were assessed using *T* tests or Mann Whitney when appropriated. Categorical comparisons were assessed using *x*^*2*^-square test. Spearman’s correlation as used to examine the relationship between change in serum markers of bone turnover and time after procedure. All statistical analyses were performed using SPSS (Statistical Package Social Sciences) software version 18 for Windows (Illinois, MA, USA). A *p* value ≤ 0.05 was considered statistically significant.

## RESULTS

After exclusion of missing data on nutritional assessment, biochemical and hormonal parameters, 61 BS (n = 58 RYGB and n = 3 BD-DS) and 30 Cont were available for enrollment in the study. The median [IQR] post-surgical time was 4 [1-7] years and the mean decrease in BMI after surgery was 36% (data not shown in table). As shown in Table 1, no significant differences were observed regarding gender distribution and mean age between BS and Cont. The mean values of BMI, daily dietary intake of energy, carbohydrate, protein and sodium chloride (estimated from 24-hour urinary sodium, for better accuracy) were significantly lower in the BS compared to Cont. Daily consumption of calcium and lipids did not differ between groups. The mean serum BAP was significantly higher for BS versus Cont patients and had a positive correlation with time after procedure (r = 0,278, p = 0,04). Serum leptin was lower in BS group compared to Cont and no significant differences were found in eGFR, ionized calcium, albumin, 25(OH)D, PTH and FGF-23 between groups. Urinary DPYD was higher, calcium and sodium were lower in BS versus Cont.

**Table 1:** Nutritional data, serum and urinary parameters

Parameter	Cont [n = 30]	BS [n = 61]	p
Gender (F/M)	23/7	51/9	0.52
Age (years)	48.0 ± 8.8	47.1 ± 9.9	0.69
eGFR	102 [85-111]	104 [97-109]	0.32
BMI [kg/m^2^]	46.5 ± 7.3	31.5 ± 6.0	< 0.001
Energy [kcal]	1739 [1182-2743]	1272 [931-1714]	0.01
Dietary carbohydrate [g]	199 [149-390]	142 [106-192]	< 0.001
Dietary protein [g]	75 [62-100]	51 [39-70]	< 0.001
Dietary lipids [g]	45 [27-57]	53 [32-76]	0.27
Dietary calcium [mg]	502 [395-1278]	517 [324-740]	0.32
Dietary NaCl [g]	11 [7-15]	8 [6-11]	0.01
Serum ionized Calcium [mmol/L]	1.3 ± 0.0	1.3 ± 0.0	0.15
Serum Albumin [g/dL]	4.2 ± 0.2	4.2 ± 0.2	0.80
Serum 25(OH)D [ng/mL]	21.0 [17-24]	19.1 [14-26]	0.51
Serum PTH [pg/mL]	61.0 ± 22.7	61.0 ± 23.8	0.98
Serum FGF-23 [kRU/mL]	41.1 [27-53]	44.4 [16-58]	0.53
Serum BAP [U/L]	17.9 ± 5.6	24.2 ± 9.6	0.003
Serum Leptin [ng/mL]	44.7 ± 11.6	32.2 ± 14.6	< 0.001
Urinary DPYD [nmol/mmol creat]	5.1 [3-6]	5.6 [3-9]	0.041
Urinary calcium [mg/24h]	161 [23-488]	89 [21-270]	0.001
Urinary sodium [mg/24h]	184 [122-252]	141 [105-181]	0.01
Urinary creatinine [mg/24h]	1474 ± 511	1112 ± 302	0.000

Data are presented as mean ± SD or median [interquartile interval]. Cont: control group, BS: bariatric surgery, eGFR: estimated glomerular filtration rate, BMI: body mass index, PTH: parathyroid hormone, FGF-23: fibroblast growth factor-23, BAP: bone alkaline phosphatase, DPYD: deoxypyridinoline.

## DISCUSSION

The main finding of the present study was an increased bone turnover, disclosed among bariatric patients in the late postoperative period when compared to morbidly obese individuals.

Several investigators have reported bone mineral metabolism disturbances following bariatric surgery, including either reduced BMD (8,11-13) or alterations in bone biochemical markers of bone turnover (14,15). Although hypovitaminosis D, secondary hyperparathyroidism and mechanical unloading of the skeleton due to weight loss have been implicated, the underlying mechanisms responsible for such changes are not fully elucidated.

In the present study, median serum 25(OH)D in BS group was low and not different from Cont before surgery, which agrees well with other reports (9,12). Whereas hypovitaminosis D in class II and III obese patients more often occurs because of the sequestration of vitamin D in adipose tissue (16), the reasons in BS patients include not only pre-operative low levels of 25(OH)D (15), but also low compliance with oral supplements (17). In the current series, despite of the low calcium consumption besides hypovitaminosis D, BS group did not present a higher median serum PTH. Nevertheless, the presence of secondary hyperparathyroidism among bariatric patients is still a matter of debate since some investigators reported normal levels of PTH (8,13,15), while others did not (14,18,19).

The significantly higher serum BAP levels in BS than Cont patients observed in the present study suggests the presence of increased bone formation, in agreement with the data reported by Bruno and cols. (15) and with other investigators, who employed different biochemical markers of bone formation (8,12). Moreover, urinary DPYD was also higher in BS patients than in Cont, indicating an increased bone resorption, as detected by other investigators (20,21), even when using different bone resorption markers as well (8,20,22,23). Therefore, an increased bone turnover has been clearly evidenced by the present findings, which corroborates with numerous reports (8,12,13,15,24), although not with all of them (9,21).

We observed a low calcium intake by BS patients (similarly to Cont), as observed by Menegati and cols. (19), what may be a caution against dumping (25). Inadequate calcium intake may contribute to impair bone homeostasis, due to increases in serum PTH and 1,25-dihydroxyvitamin D3 (1,25(OH)_2_D_3_) (26), and a high sodium consumption may also compromises bone health (27,28). Furthermore, the presence of reduced intestinal calcium absorption even under conditions of both calcium and vitamin D supplementation in bariatric patients (11) may render bone condition even worse. The observed increased bone turnover in BS group could be accounted for by a negative calcium balance. However, given that urinary calcium and sodium were significantly lower in BS versus Cont, the contribution of negative calcium balance did not seem to be important.

The present sample consisted of bariatric patients with a median follow-up of 4 years after the procedure with a handful of them attaining more than 7 years of follow-up. Of note, we found a positive correlation of serum BAP with time after surgery. A substantial increase in bone formation and resorption markers had occurred up to 24 months after gastric bypass despite weight stability, as described by many authors (12,13,29), reinforcing that bone remodeling remains elevated despite weight loss plateaus after the first year and are not exclusively due to mechanical unloading of the skeleton.

It is well recognized that the adipose tissue synthesizes and releases a series of hormones and adipokines involved in bone metabolism through direct effects on bone formation and resorption (30). When compared to Cont, bariatric patients from the current series presented a lower mean value of serum leptin, one of the most important cytokines derived from fat tissue, which acts on hypothalamic neurons by stimulating sympathetic postganglionic neurons via axons that enter the bones to inhibit bone formation (31,32). Our findings are in agreement with other reports after bariatric surgery (13,15). Bruno and cols. (15) also showed that a reduction in leptin represented an independent predictor of increase in N-teloptide of type 1 collagen (NTX), a bone resorption marker, among bariatric patients. A more recent study of bone structural changes evaluated through high-resolution peripheral quantitative computed tomography by Frederiksen and cols. (13) revealed an inverse association between leptin and trabecular number. Although there is still some controversy regarding the correlation of leptin with BMD (30), Thomas and cols. (33) noticed a direct association between leptin and BMD only among women, in the general population, suggesting that leptin could mediate at least part of the protective effect of fat mass on the skeleton. However, the reciprocal action between bone and adipose tissue is complex and the underlying mechanisms by which leptin regulates bone remodeling remain not fully elucidated (34). In an experimental study, Tsuji and cols. (35) showed that leptin increased FGF-23 expression in leptin-deficient ob/ob mice, acting directly on bone cells, and suppressed renal 1,25(OH)_2_D_3_ synthesis. Conversely, one may speculate that the reduction of leptin following bariatric surgery could be associated with an increased 1,25(OH)_2_D_3_, hence contributing to stimulate bone resorption, especially under conditions of low calcium consumption (26). However, in the current series, median values of serum FGF-23 did not differ between groups, even in the presence of lower serum leptin in BS versus Cont. Moreover, levels of 1,25(OH)_2_D_3_ were not determined. Other limitations of the present study included the lack of BMD and body composition data from both groups and the absence of enough test results of mineral metabolism parameters among bariatric patients before the surgery, to allow us to make comparisons before and after the procedure.

In summary, BS patients exhibited significantly higher serum BAP, urinary DYPD and lower serum leptin levels than Cont, without statistical significance in circulating levels of 25(OH)D and PTH, indicating that other mechanisms, not solely associated to the calciotropic hormones, might be operating to explain the increased bone turnover after bariatric surgery. Such findings are in agreement with other investigators (14,18) and according to Bruno and cols. (15), even the supplementation of vitamin D was not capable to prevent the increase in bone turnover seen after bariatric surgery.

In conclusion, the present study showed an increase in bone markers of both bone formation and resorption among bariatric patients up to more than 7 years after the surgical procedure, suggesting that an increased bone turnover persists even at a very long-term follow-up in such patients. Additional studies are still warranted to better elucidate the underlying mechanisms for these findings, aiming to mitigate derangements in bone metabolism and helping to delineate earlier interventions.
